# Two novel variant allele of the RHD gene detected in two Chinese blood donors with the RHD negative phenotype

**DOI:** 10.1111/tme.70001

**Published:** 2025-07-16

**Authors:** Qiuhong Mo, Xuejun Liu, Mingshuang Lai, Rongji Lai, Limin Chen, Baoren He

**Affiliations:** ^1^ Chinese Academy of Medical Sciences and Nanning Blood Center, Nanning Blood Center Joint Laboratory on Transfusion‐Transmitted Diseases (TTDs) between Institute of Blood Transfusion Nanning China; ^2^ Nanning Institute of Transfusion Medicine Nanning Blood Center Nanning China

**Keywords:** *RHD* blood group, *RHD* genotyping, *RHD* variant

## Abstract

**Background:**

The D antigen is the most clinically important antigen in the Rh system due to its high immunogenicity and being the main cause of hemolytic disease of the foetus and newborns (HDFN). High polymorphism of the *RHD* gene implicates that new *RHD* variant alleles may be regularly identified.

**Study Design and Methods:**

D antigen status was examined using saline tubes with IgM anti‐D (clone RUM‐1). If a negative result was observed, indirect antiglobulin test (IAT) and adsorption‐elution test were performed with four monoclonal anti‐D reagents. A D‐screen identification kit was used for partial D identification. All 10 exons of the RHD gene plus flanking intronic regions were amplified and sequenced.

**Results:**

The serological investigation showed clearly negative results with all anti‐D reagents used when testing against both probands RBCs. The sequencing results of probands revealed c.802‐1G>C mutation in proband 1 and c.1154‐2A>T mutation in proband 2. These mutations resulted in the change of 3′ splice site in the intron‐exon junction of intron 5 or intron 8 of the *RHD* gene from AG to AC or TG, abolishing the splicing effect.

**Conclusions:**

We identified two novel splicing variants resulting from c.802‐1G>C and c.1154‐2A>T mutations in the *RHD* gene. Both mutations may abolish the splicing effect of the involved exons, leading to the D‐negative phenotype.

## 1 INTRODUCTION

In the 47 human blood type systems currently detected, Rhesus (Rh) is an important and most complex blood system in clinical transfusion, second only to ABO, containing over 50 RH antigens.^1^ Among them, D antigen has the strongest immunogenicity and the most clinical significance, which can cause serious transfusion reactions and hemolytic disease of the foetus and newborns (HDFN). High polymorphism in D antigen indicates that new *RHD* variant alleles may be regularly identified.^2^ In fact, more than 90 D‐negative alleles involving various mutational mechanisms have been identified, according to ISBT allele nomenclature version v6.4 (http://www.isbtweb.org/working‐parties/red‐cell‐immunogeneticsand‐blood‐groug‐terminology).

In this study, we report two novel variant alleles of the *RHD* gene detected in two Chinese blood donors with D‐negative phenotype. Both variants were caused by point mutations at the intron‐exon junction, leading to the loss of splicing effect of the exons.

## 2 MATERIALS AND METHODS

The two probands are female blood donors in Nanning blood centre. Routine serological testing was performed by saline tubes with IgM anti‐D (clone RUM‐1). Because the results of routine testing were negative, indirect antiglobulin test (IAT) and adsorption‐elution test were performed with four monoclonal anti‐D reagents: Novaclone 175‐2/D415 1E4 (R1) (Immucor, Inc., Peachtree Corners, GA); TH‐28/MS‐26 (R2) (Millipore Ltd., Watford, UK); TH‐28/MS‐26 (R3) (Sanquin Reagents, Amsterdam, Netherlands); and MS‐26 (R4) (Shanghai Hemo‐Pharmaceutical& Bio, Shanghai, China). RhC/c and RhE/e antigens were assayed using standard hemagglutination tube methods with anti‐C, ‐c, ‐E, and ‐e IgM monoclonal antibodies, respectively (Shanghai Hemo‐Pharmaceutical and Biological Inc., Shanghai, China). The absorption‐elution test using the acid elution method was performed to detect weak D antigen expression. Briefly, 200 μL packed RBCs were washed twice with 0.9% saline and they were incubated with monoclonal anti‐D reagents (R1/R2/R3/R4) at 37°C for 1 h. After centrifugation and saline wash for 5–6 times, acid elution releases bound antibodies, which are neutralised (blue‐purple endpoint) and tested via anti‐human globulin card with D+ RBCs (37°C, 15 min). RhD+ (ccDEE) and RHD‐negative (ccdee)were used as controls. A D‐screen identification kit was used for partial D identification (DIAGAST, Loos, France). *RHD* genotyping was performed on DNA isolated from a peripheral blood sample with a commercially available extraction kit (QIAamp DNA Blood Mini Kit). *RHD* variants and zygosity were assessed by commercially available SSP‐PCR kits (Tianjin Super Biotechnology Development Co., Ltd., Tianjin, China). All 10 exons of the *RHD* gene plus flanking intronic regions were amplified by polymerase chain reaction and sequenced as previously described.^3^ The possible impact of the mutations on protein structure and function was predicted by MutationTaster (http://www.mutationtaster.org/)^4^ and varSEAK (https://varseak.bio/).^5^ This study was approved by the Medical Ethics Committee of the Nanning Blood Centre (approval number: 2024‐07‐01).

## 3 RESULTS AND DISCUSSION

**FIGURE 1:**
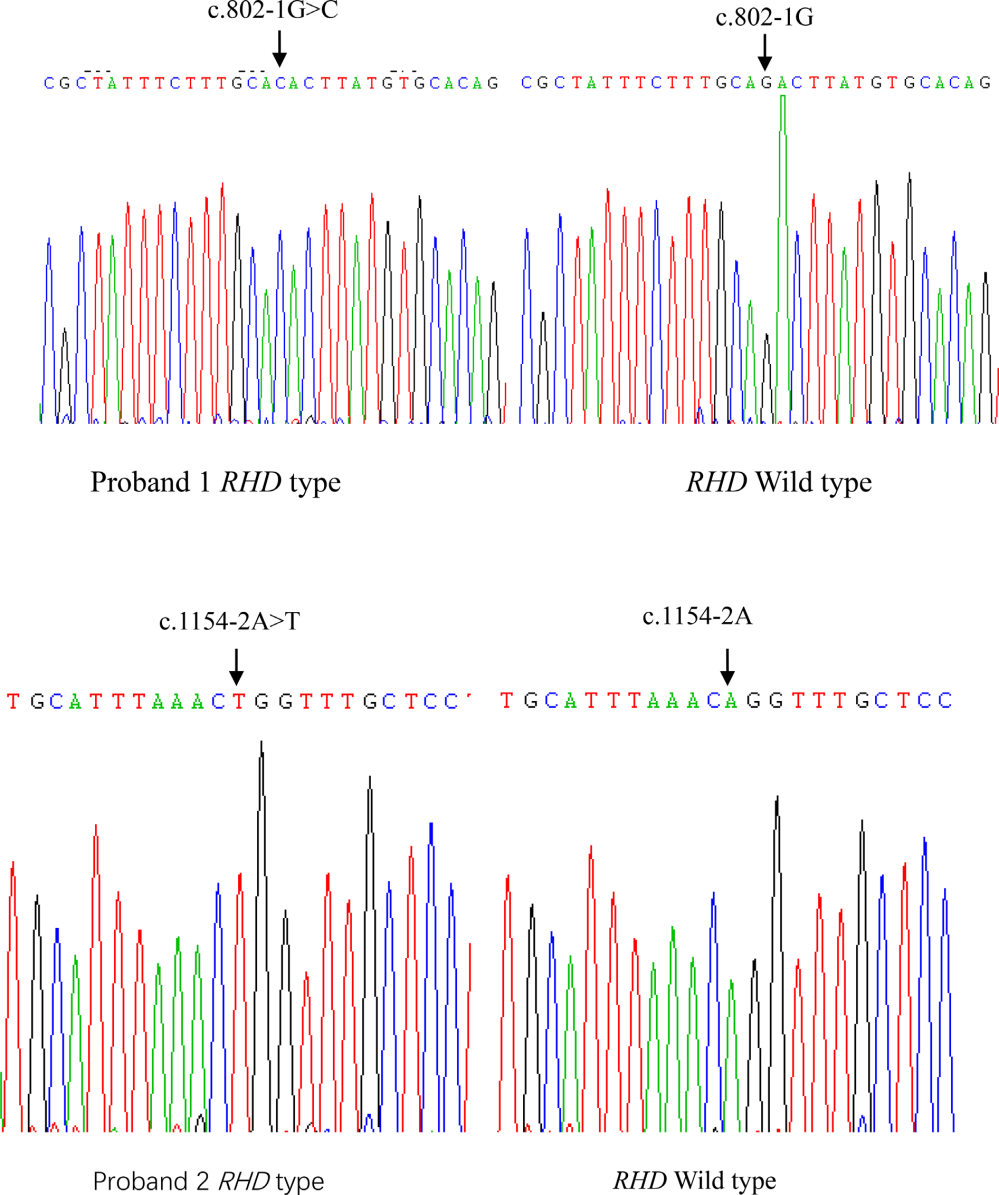
Sequencing results of exon 6, exon 9 and flanking intronic regions of RHD gene.

The serological investigation showed clearly negative results with all anti‐D reagents when testing against both probands RBCs. The absorption‐elution tests with four monoclonal antibodies for both probands yielded negative results under strict positive and negative controls. The RhCcEe phenotype of the probands were found to be Ccee. Results of serological and *RHD* gene analysis are summarised in Table 1. *RHD* zygosity testing confirmed that both probands were hemizygous (*RHD/RHD* deletion). *RHD* gene analysis by SSP‐PCR kits did not reveal any common variant allele. Sequencing analysis revealed that exons 1–10 and the flanking regions of the *RHD* gene in both probands were identical to the wild type reference (NG_007494.1), except for the c.802‐1G>C mutaion in proband 1 and c.1154‐2A>T mutation in proband 2 (Figure 1). The mutations at the 3 ‘end of the intron‐exon junction of intron 5 or intron 8 of the *RHD* gene from AG to AC or TG leading to the loss of splicing effect of the exon. Both mutations may affect the structure and function of RHD protein because the PhastCons scores of the splice site change were predicted to be 0.873 and 0.984 respectively by Mutation Taster. The score ranges from 0 to 1, with higher scores indicating more impact on the structure and function of the involved protein. Interestingly, both mutations cause the respective exon to lose the splicing effect as predicted by VarSEAK. The sequences of these two novel *RHD* alleles have been deposited to GeneBank with Accession Number ON820228 and OR426989. While this study was limited by the lack of mRNA sequencing validation, existing reports^1^, ^4^, ^6^, ^7^ suggested that mutations at exon‐intron junctions of the *RHD* gene could disrupt mRNA processing, leading to weak D or D‐negative phenotypes. Consistent with these studies, we speculated that the c.802‐1G>C and c.1154‐2A>T mutations in the *RHD* gene may cause mRNA splicing defect resulting in the observed RhD‐negative phenotype in both probands.

**TABLE 1:** Results of serological and *RHD* gene analysis.

	Indirect antiglobulin test					D‐screen											
Member	R1	R2	R3	R4		1	2	3	4	5	6	7	8	9	RhCE phenotype	*RHD* genotype	
Proband 1	0	0	0	0		0	0	0	0	0	0	0	0	0	Ccee	D^c.802‐1G>C^/RHD deletion	
Proband 2	0	0	0	0		0	0	0	0	0	0	0	0	0	Ccee	D^c.1154‐2A>T^/RHD deletion	

*Note*: The anti‐D clones of the D‐screen identification kit are No. 1‐HM10(IgM), No. 2‐HM16(IgG), No. 3‐P3X61(IgM), No. 4‐P3X35(IgG), No. 5‐P3X21211F1(IgM), No. 6‐P3X21223B10(IgM), No. 7‐P3X241(IgG), No. 8‐P3X249(IgG), and No. 9‐P3X290(IgG).

## 4 CONCLUSIONS

In conclusion, we identified two novel *RHD* alleles with c.802‐1G>C and c.1154‐2A>T mutations that are likely responsible for the D negative phenotype. Both mutations are located at the splicing sites of introns 5 or 8 of the RHD gene, causing them to lose the splicing effect, leading to the D negative phenotype.

## AUTHOR CONTRIBUTIONS

All listed authors have contributed to the manuscript substantially. Qiuhong Mo: Conceptualising, supervision, methodology, writing – original draft and editing, funding acquisition, investigation. Xuejun Liu: Conceptualising, validation, investigation, funding acquisition. Mingshuang Lai & Rongji Lai: Conceptualising, investigation. Limin Chen: Conceptualising, writing – review and editing. Baoren He: Conceptualising, methodology, investigation, data curation, formal analysis, funding acquisition, writing – original draft, writing – review and editing.

## FUNDING INFORMATION

This work was funded by Nanning Science and Technology Development Plan projectof Nanning Science and Technology Bureau (20173157‐8).

## CONFLICT OF INTEREST STATEMENT

The authors have no competing interests.

## PATIENT CONSENT STATEMENT

Both probands are adults, and informed consent was obtained from them.

## Data Availability

The data that support the findings of this study are openly available in [Nucleotide] at [https://www.ncbi.nlm.nih.gov/nuccore/], reference number [ON820228 and OR426989, respectively].
